# A New Multichannel Spectral Imaging Laser Scanning Confocal Microscope

**DOI:** 10.1155/2013/890203

**Published:** 2013-03-26

**Authors:** Yunhai Zhang, Bian Hu, Yakang Dai, Haomin Yang, Wei Huang, Xiaojun Xue, Fazhi Li, Xin Zhang, Chenyu Jiang, Fei Gao, Jian Chang

**Affiliations:** Suzhou Institute of Biomedical Engineering and Technology, Chinese Academy of Sciences, Suzhou 215163, China

## Abstract

We have developed a new multichannel spectral imaging laser scanning confocal microscope for effective detection of multiple fluorescent labeling in the research of biological tissues. In this paper, the design and key technologies of the system are introduced. Representative results on confocal imaging, 3-dimensional sectioning imaging, and spectral imaging are demonstrated. The results indicated that the system is applicable to multiple fluorescent labeling in biological experiments.

## 1. Introduction

As an effective and necessary scientific instrument for observing small structures, laser scanning confocal microscopy has been widely applied in tissue biology, cell biology, molecular biology, genomics, embryology, neurology, embryology, pathology, immunology, epidemiology, oncology, bacteriology and virology, and so forth [1–4]. The confocal system uses the spatial filter techniques to eliminate the out-of-focal-plane light. Only the light from the focal plane can be detected. Hence, the system has high spatial resolution and signal-to-noise ratio [5]. At the same time, it has the capability of optical sectioning in *z* axis direction, which enables 3-dimensional imaging of thick samples [6].

The confocal concept was proposed by Minsky in 1950s [7]. The first commercial product was released in 1987. After that, the confocal techniques have attracted researchers' attention, and new developments in both research and products were achieved. To date, various aspects of the system have been investigated to improve the image resolution [8, 9], and products with new functions have been launched in the market.

 Currently, there is a need to image multiple fluorescent labeling tissues simultaneously, discriminate the various fluorescent components [10], and implement complex functional experiments, such as fluorescence recovery after photobleaching (FRAP) [11], fluorescence resonance energy transfer (FRET) [12], and fluorescence lifetime imaging microscopy (FLIM) [13]. We thus developed a new multichannel spectral imaging laser scanning confocal microscope to meet these demands. The following sections describe the methodology of the system and demonstrate representative experimental results on the system.

## 2. System Design

The hardware of multi-channel spectral imaging laser scanning confocal microscope consists of a fluorescence microscope, a confocal scanning head, a laser source, an electrical control box, and a computer. Modular design is used in the system; the confocal scanning and multi-channel spectral imaging are integrated into the scanning head; the multiple lasers are placed in a laser cabinet; the scanning head and laser cabinet are separated to avoid laser vibration effects on the confocal imaging; the complex electrical control parts are integrated into an electrical control box; the control software is installed in the computer.

 The fluorescence microscope has an independent illumination system (high-pressure mercury lamp for epi-illumination and tungsten halogen lamp for transmission illumination) and an optical path for visual observation. The optical path for visual observation is used for initial observation and localization of the sample before switching to the confocal system. The confocal system also has an independent illumination system (multiple lasers) and a detection system (PMTs). The 2-dimensional optical sectioning, 3-dimensional imaging, and selected spectral bandwidth imaging can be implemented through control software. 

**Figure 1:**
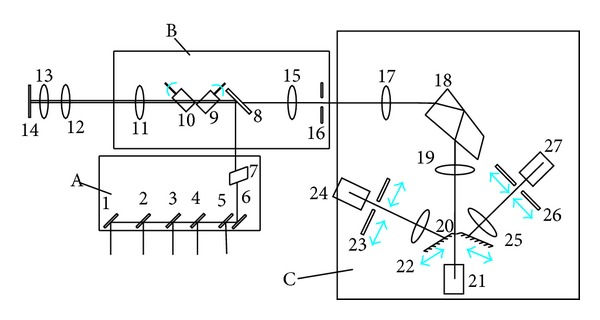
Diagram of the confocal scanning head. A: Laser combination and selection module, B: confocal scanning imaging module, C: multi-channel spectral imaging module, 1: dichroic mirror for spare laser combination, 2–5: dichroic mirror for four lasers combination, 6: mirror, 7: acousto-optical tunable filter (AOTF), 8: dichroic mirror for excitation and fluorescence separation, 9-10: *X-Y* galvanometer optical scanner, 11: scanning lens, 12: tube lens, 13: objective lens, 14: sample slide, 15: pinhole lens, 16: pinhole, 17: collimation lens, 18: prism, 19: focal lens, 20: movable slit 1, 21, 24, 27: PMTs, 22, 25: relay lens, 23, 26: movable slit 2, 3.

**Figure 2:**
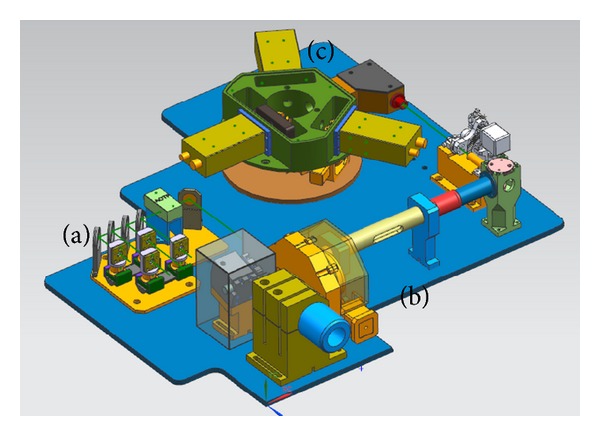
Internal composition of the confocal scanning head.

Figure 1 is the diagram of the confocal scanning head, which is the key component of the confocal microscopy. The internal composition is shown in Figure 2. It can be divided into three parts: (a) laser combination and selection module, (b) confocal scanning imaging module, and (c) multi-channel spectral imaging module.

 In the laser combination and selection module, the four fiber-coupled semiconductor lasers (405 nm, 488 nm, 561 nm, and 638 nm) are combined into one beam using dichroic mirrors, then the combined beam goes through the AOTF. The combined beam's laser wavelength and power can be adjusted by changing the frequency and amplitude of the ultrasonic wave. The spare space is reserved for the additional laser extension for the future upgrade. 

 The confocal scanning imaging module is mainly composed of scanning lens, *X-Y* galvanometer optical scanner, dichroic mirror dial, pinhole lens, and three aperture-variable pinholes. The laser is reflected from the dichroic mirror assembly and steered by the *X-Y* galvanometer optical scanner. Then, it goes through the scanning lens and is focused on the sample by the objective lens. The excited fluorescence is recollected by the same objective lens, goes through the scanning lens and dichroic mirror assembly, and is focused at pinhole by the pinhole lens. There is an individual dichroic mirror assembly (including a dichroic mirror and a fluorescence filter) for each laser wavelength. For the multiple lasers, there is another dichroic mirror assembly. An aperture-variable pinhole is for 60x oil immersion objective lens. The second one is for 100x oil immersion objective lens. The third one is used to balance image resolution and signal-to-noise ratio.

 The multi-channel spectral imaging module is based on light separation of the prism. There are three spectral detection channels. The spectrum of the fluorescence is expanded by the prism's dispersion. At the focal plane of the fluorescence, there are two movable mirrors. Part or all of the fluorescence passes through the slit between the two mirrors and is collected by the first spectral channel. The fluorescence can also be reflected by the two movable mirrors and then goes to the second and third spectral detection channels individually. There are two movable diaphragms in the second and third spectral channels, which can be used to control the spectral band width of the fluorescence by adjusting the location and the size.

## 3. Key Technologies

The multi-channel spectral imaging laser scanning confocal microscopy involves several key technologies, such as high-speed synchronization control of galvanometer scanning, 3-dimensional optical sectioning imaging, multi-channel spectral imaging, spatial fine pinhole filter technique [4], and optimization of the point spread function for illumination [8]. The first three are the foundation of the confocal microscopy and will be discussed in the follow paragraphs. 

### 3.1. High-Speed Synchronization Control of Galvanometer Scanning

**Figure 3:**
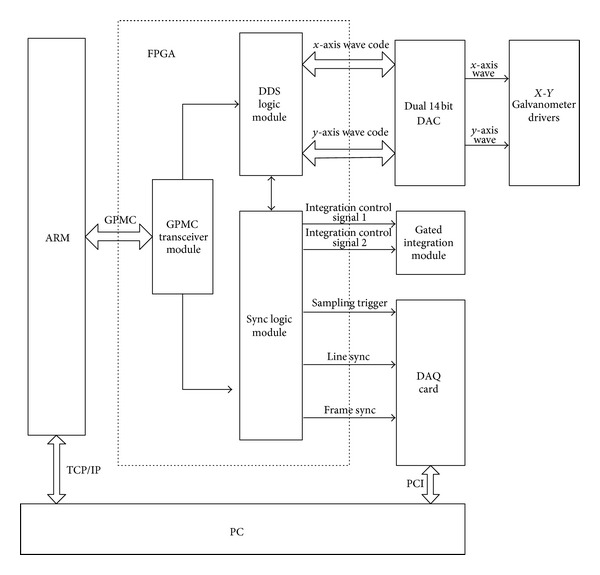
Diagram of the galvanometer synchronization control system.

The scanning control system is responsible for the control signal generation for the galvanometer. The data acquisition and hardware synchronization are realized by the combination of sampling signal control system and galvanometer scanning control system. It ensures the accuracy of scanning data for the image reconstruction.  Figure 3 is the diagram of the galvanometer synchronization control system. ARM accomplishes the function of user interface, calculation of waveform data, parameters' setup for sampling signal, and writing operation to FPGA. FPGA module realizes the function of DDS signal generation and sampling control signal generation. DAC transforms the output of the FPGA to the analog voltage signal, which is used to control the movement of the galvanometer scanner.

**Figure 4:**
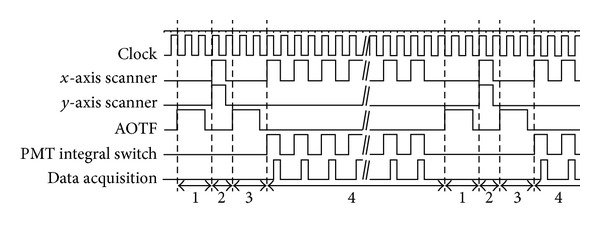
Synchronization between the galvanometer scanner control and data acquisition. Phase 1: AOTF is switched off after one line scanning, phase 2: *x*-axis and *y*-axis scanners retrace to the start position for the next line scanning, phase 3: AOTF is switched on, phase 4: process of one line scanning.

 The strict synchronization between the galvanometer scanner and data acquisition is very important. In the fast retracing scanning process, data is not collected. To avoid the photobleaching of fluorescence, AOTF is used for rapid switch off of laser [14]. The synchronization between the galvanometer scanner control and data acquisition is shown in Figure 4. The requirement for the synchronization is as follows: (i) frame synchronization signal (the rising edge of the square wave). It has the same frequency as the slow scanning trigger signal. It appears on the rising edge of the square wave during the slow scanning process. (ii) Line synchronization signal. The data is collected whenever the trigger voltage is at the high level. The whole line data are obtained at each high level trigger signal. It has the same frequency as the fast scanning trigger signal. The trigger signal is at high level when it is in scanning, while it is at low level when it is in the retracing process. (iii) Sampling pulse. The sampling pulse is generated when the line synchronization signal is high. The sampling control signal is synchronized with galvanometer control voltage from the DAC.

### 3.2. 3-Dimensional Optical Sectioning Imaging

One feature of the confocal microscopic imaging is that only the very thin tissue in the focal plane can be imaged once a time. The tissue above or below the focal plane cannot be imaged due to the block of the pinhole. This feature makes the confocal microscopic imaging suitable for the lossless sectioning imaging. After collecting a series of 2-dimensional images, 3-dimensinal image of the tissue can be reconstructed. 

**Figure 5:**
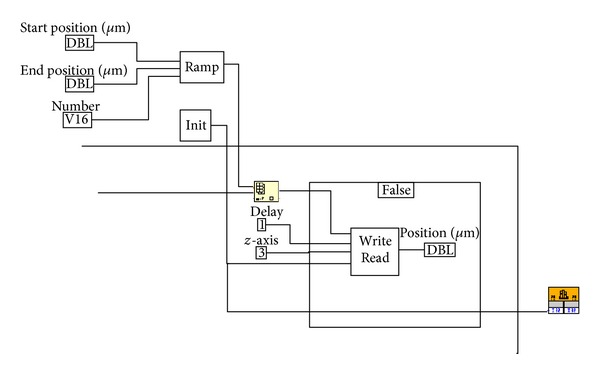
Control program of nanopositioner using LabVIEW.

 During the experiment, the sample tissue needs to be moved accurately in the *z* direction with the step size of 0.2 *μ*m. A piezo nanopositioner meets the requirement. The parameters such as single step size and total step numbers can be adjusted by the control software. Those parameters are the basis of the 3-dimensional confocal images.  Figure 5 demonstrates the control program of nanopositioner using LabVIEW. Firstly, the positioner is initialized according to the setting of start position, end position, and number of slices. Then, the positioner is moved to the start position, and the movement of each slice is calculated. It is kept in the current position during the process of 2-dimensional scanning. After that, it is moved to the next slice and a new 2-dimensional scanning starts. The process is sequentially cycled until finishing the number of slices. During the process, the interface shows the actual position of the nanopositioners. 

 In order to get a series of 2-dimensional confocal images, a relatively long time is needed. During the time, the sample slide should be fixed, which requires that the supporting structure for the slide and microscope base have enough rigidity. However, it is still hard to eliminate all the small drifts during the long data acquisition time. So, postprocessing algorithms such as image registration are needed to correct the drifts.

 The 3-dimensional image is reconstructed from a series of 2-dimensional images using volume rendering. The volume rendering method resamples the collected 3-dimensional data and transforms the data into 2-dimensional discrete signals in image display cache. It classifies the 3-dimensional data, sets color and opacity for each voxel, and generates the 2-dimensional display image according to the illumination and shading model. Main algorithms of volume rendering include ray casting, footprint tracing, and shear deformation. The ray casting is used in our system.

### 3.3. Multichannel Spectral Imaging

In the case that multiple fluorescence is excited simultaneously, single channel or multi-channel detection is needed to effectively differentiate the fluorescence components. Fluorescence efficiency, spectral resolution, dispersion linearity, and stray light effect need to be considered in the design.

 The fluorescence usually is weak for biological samples. So, the light transmission efficiency is very important for the design of the fluorescence spectrometer. In the visible spectrum, the maximum efficiency of the grating spectrometer is only 1/2–2/3 of that of prism spectrometer. The efficiency decreases to 1/4 of that of prism spectrometer when it is far away from the blazed wavelength. Hence, grating spectrometer loses more fluorescence for the weak signal detection.

 In the confocal system, the grating area used is very small and the line number is at the level of 10^3^. The theoretical resolution is higher than prism. However, the required minimum spectral band width is only 5 nm. The high spectral resolution of the grating spectrometer is not fully utilized.

 Compared with linear dispersion of grating spectrometer, dispersion of prism spectrometer is nonlinear. In the confocal system, the user needs to select the spectral band before data acquisition. So the nonlinear dispersion has little effect on the imaging system. 

 Stray light of prism spectrometer mainly comes from prism surface reflection. After antireflection coating on the prism surface, stray light decreases to 10^−3^ or less. For the grating spectrometer, stray light comes from many sources such as grating diffraction energy level overlap, 30–40% unused energy from zero order, and high orders. The non-working surface of the grating groove and ghost lines caused by grating ruling error also contributes to the generation of stray light.

 So, the prism spectrometer outperforms the grating spectrometer and is thus used in the system.

 Due to the cost limitation, there are 3 fluorescence detection channels in the system. It can be extended to have more channels as desired. The multi-channel spectral imaging system based on prism is shown in Figure 1. The spectral range of detection of each channel can be adjusted from 400 to 700 nm. The minimum spectral band width is 5 nm and the maximum band width is 300 nm. 

 In the system, constant deviation angle is used to expand the spectrum. The optical path is rotated 90 degrees to make sure tha the total deviation angle is constant after the prism for the light in the minimum deviation condition. The dispersion function of constant deviation angle of the prism is equal to the prism with the apex angle of 60 degrees.

 The fluorescence from the sample is focused on the image plane after collimation. The prism is fixed, and the detected central wavelength and spectral band width can be adjusted by the exit slit assembly. In Figure 1, the exit slit 1 is located at the focal plane. The two blades of the slit, coated with reflective film, are tilted to both sides by 30 degrees independently. The light reflected from the blades' surface goes to the channel 2 and 3. Slit 1 controls the spectral band width detected in PMT 1. Slit 2 and 3 adjust the spectral band width detected in PMT 2 and 3 separately. 

## 4. Experiment Results

**Figure 6:**
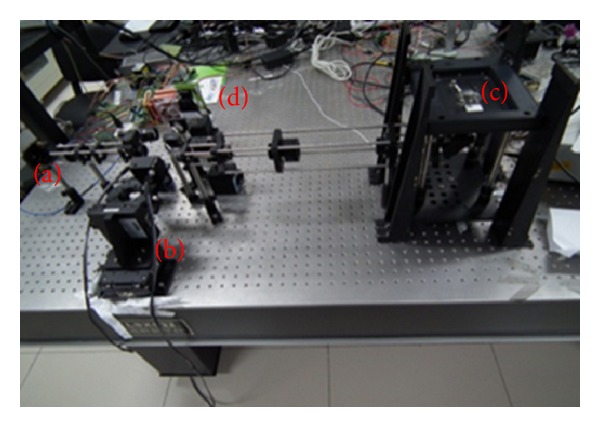
Confocal microscopic imaging system setup.

Figure 6 shows the system setup. (a) is the laser source, (b) is the *X-Y* galvanometer optical scanner, (c) is the nanopositioner, and (d) is the detector. In the system, oil immersion objective UPLSAPO 100x from Olympus was used. The NA is 1.4 and working distance is 0.13 mm. Super apochromat objectives fully compensate for both spherical and chromatic aberrations from the UV to the near infrared region. The illumination source was semiconductor fiber laser from Pavilion Integration Corporation. The fibers are single-mode polarization maintaining. The wavelength of lasers are 405 nm, 488 nm, 561 nm, and 638 nm. The output power is 35 mw. The optical scanner 6215H from Cambridge Technology was used. The PMT was H10720 from Hamamatsu. Despite the small size nearly equal to photodiodes, this PMT delivers high gain, wide dynamic range, and high-speed response. PCI 6132 from National Instruments was used as data acquisition board. It has 4 simultaneously sampled analog inputs, up to 2.5 or 3 MS/s in warp mode. The ADC resolution is 14 bits. Experiments of the confocal imaging, 3-dimensinal sectioning imaging, and spectral imaging were accomplished based on the system. 

### 4.1. Confocal Imaging Experiment

**Figure 7:**
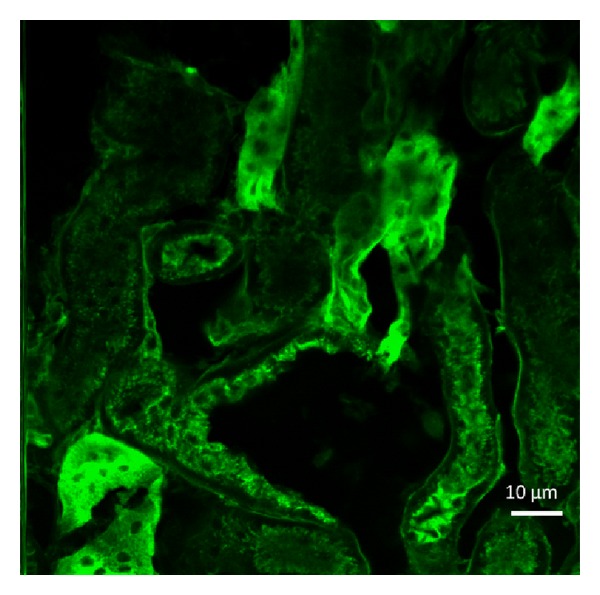
Confocal fluorescence image of rat kidney slice.

In the experiment, the illumination source was 488 nm laser. The biological tissue prepared was slide-mouse kidney section from Molecular Probes. The slide contains a 16 *μ*m cryostat section of mouse kidney stained with a combination of fluorescent dyes. Alexa Fluor 488 wheat germ agglutinin, a green-fluorescent lectin, was used to label elements of the glomeruli and convoluted tubules. The imaging area was 112 *μ*m × 112 *μ*m. The result is shown in Figure 7. The glomeruli and convoluted tubules were clearly distinguished. It can be observed that the confocal image is only from a very thin tissue section (focal plane), which is the feature of the confocal imaging. The dark area means no tissue in that section, which is totally different from traditional fluorescence microscope. The images obtained from the traditional microscope are composed of a thick section. The tissue information in the focal plane as well as that above and below the focal plane is displayed together. So, the whole image contrast is degraded.

### 4.2. 3-Dimensional Sectioning Imaging Experiment

**Figure 8:**
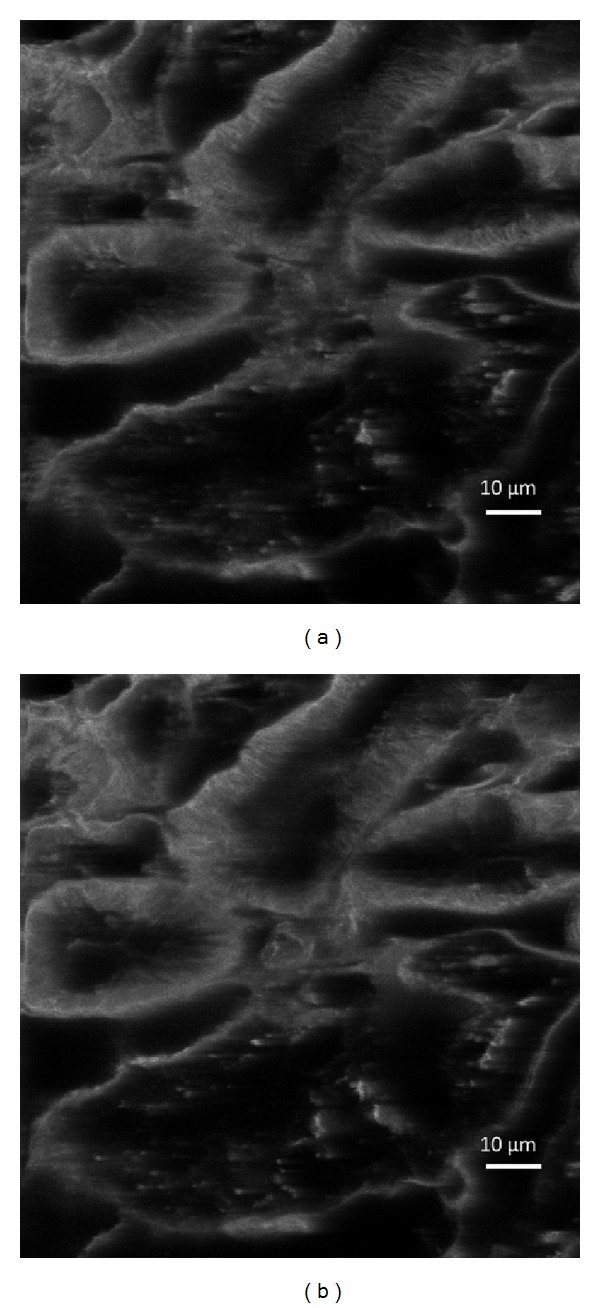
Two confocal images at various depths.

**Figure 9:**
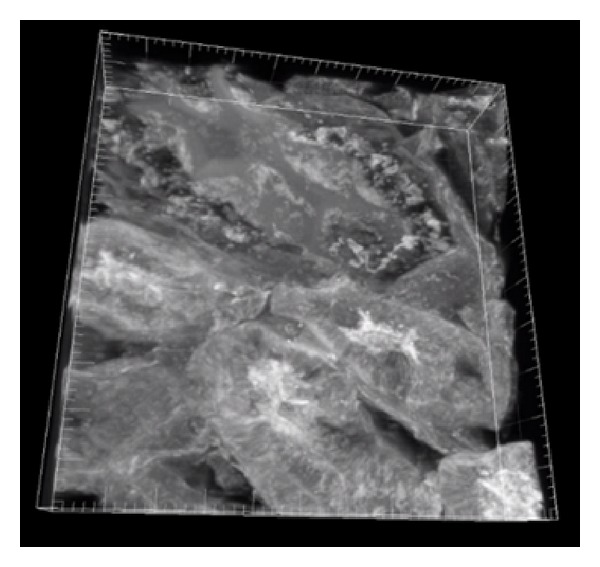
Reconstructed 3-dimensional image.

The sample and excitation source were the same as the ones used in the experiment in Subsection 4.1. The imaging area was 106 *μ*m × 110 *μ*m. To get the 3-dimensional images, a nanopositioner (Nano-Z100, Mad City Lab) was used to move the sample in *Z* direction. The step size was 0.2 *μ*m and the sample was moved 100 steps. As a result, 100 sequential images were obtained. Then the 3-dimensional image was reconstructed from those 100 images. They are shown in Figures 8 and 9. The two images of Figure 8 were focused on the two different depths in *z* position. The distance between the two images was 1.2 *μ*m. It can be observed that the details of the tissue were changed. 

### 4.3. Confocal Spectral Imaging Experiment

**Figure 10:**
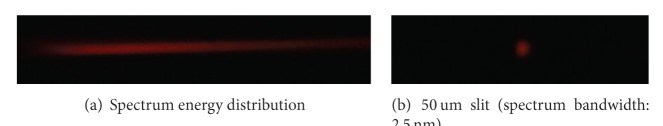
Spectrum energy distribution for Cy5.

An improved spectrometer was added before the detector and the incident slit were removed. The pinhole was imaged at incident slit and a circular slit was formed. The exit slit was replaced by a slit with adjustable position and width. The spectrum of fluorescent dye Cy5 excited by 638 nm laser is shown in Figure 10(a). The bandwidth of the spectrum was 50 nm. The fluorescence in particular bandwidth can be imaged by changing the position and bandwidth of the slit. By the calculation, the 50 *μ*m slit corresponded to 2.5 nm spectrum bandwidth. It is shown in Figure 10(b).

**Figure 11:**
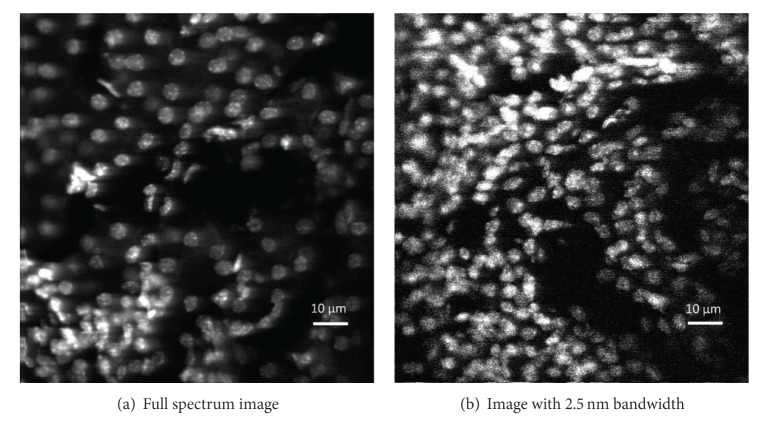
Confocal images for dye DAPI in rat kidney with various bandwidth.

 In the experiment, the illumination source was 405 nm laser. The biological tissue was rat kidney sliced from Molecular Probes. In the slice, the nuclei were counterstained with the blue-fluorescent DNA stain DAPI. Image area was 106 *μ*m × 110 *μ*m. The full spectrum image and image with 2.5 nm bandwidth are shown in Figure 11. In both images, the nuclei were clearly observed. The fluorescent light became weak and signal-to-noise ratio became worse when the spectrum bandwidth decreased. 

The system has the advantage of illumination of multiple fluorescent components simultaneously and confocal imaging of multiple detection channels. Each channel can be used for spectral imaging and spectral analysis. Prism was applied to split the spectrum, which has higher light utilization efficiency, compared to grating method. This is very important for the weak fluorescence signal. In addition, the stray light of the prism is relatively small. So the image can be obtained at higher signal-to-noise ratio. Due to the realization of multi-channel simultaneously spectral imaging, the disadvantage of the system is that many parts need to be controlled simultaneous. Hence, the control system is very complex and requires the synchronization of all parts. Besides, the real-time display of images requires intensive computation of image reconstruction and processing. The graphic workstation with high performance is needed.

## 5. Conclusions

This paper introduces our newly developed multi-channel spectral imaging laser scanning confocal microscope. The system design and key technologies (such as high-speed synchronization, control of galvanometer scanning, 3-dimensional optical sectioning imaging, and multi-channel spectral imaging) are described. The experimental results demonstrate that the system is capable of multifluorescent spectral imaging. Our multi-channel spectral imaging laser scanning confocal microscope has many advantages. Firstly, it can excite multiple dyes simultaneously and differentiate various fluorescent components effectively. In the system, four lasers of 405 nm, 488 nm, 561 nm, and 638 nm were used as illumination source. The laser source module is scalable. Additional laser extension is available for the user's upgrade. Secondly, the different spectral components from the same dye can be imaged and postprocessed to meet the requirements of biological experiments. Three fluorescence detection channels realized multiple spectral imaging simultaneously. The detected spectrum's central wavelength and spectral width can be adjusted individually for each channel. The information from the detected spectrum is fully utilized. Thirdly, the illumination and shading model was improved, and 3-dimensional image reconstruction algorithm was optimized especially for confocal sectioning images. The image reconstruction time was greatly decreased from 30 seconds to 15 seconds.
